# From Host-Derived Pressures to the Environmental Anti-Antimicrobial Peptides Resistome: Mechanisms, Reservoirs and Implications for Therapeutic Peptide Design

**DOI:** 10.3390/md24020076

**Published:** 2026-02-12

**Authors:** Yi Lu, Baomei Zhang, Zishuo Wang, Yidi He, Hezi Ge, Hongyue Ma, Pengfei Cui

**Affiliations:** 1 College of Chemistry and Chemical Engineering, Ocean University of China, Qingdao 266100, China; e1597497@u.nus.edu (Y.L.); baomeizhang@stu.ouc.edu.cn (B.Z.); 2 Haide College, Ocean University of China, Qingdao 266003, China; 15833233706@163.com (Z.W.); heyidi@stu.ouc.edu.cn (Y.H.); ghz@stu.ouc.edu.cn (H.G.); 3 Lab of Environmental Health and Ecological Engineering, College of Marine Life Science, Ocean University of China, Qingdao 266003, China

**Keywords:** antimicrobial peptides, AMP resistance, anti-AMP resistome, host-microbe co-evolution, marine and environmental microbiomes

## Abstract

Antimicrobial peptides (AMPs) are increasingly promoted as alternatives or complements to conventional antibiotics, yet growing evidence demonstrates that resistance to AMPs is neither rare nor incidental. Here, we define the anti-AMP resistome as a coordinated network of genetic, regulatory, and physiological mechanisms that enable bacteria to tolerate or evade AMP-mediated stress. We synthesize advances in understanding how envelope remodeling, efflux and sequestration, extracellular proteolysis, biofilm-associated buffering, and inducible stress responses collectively shape AMP susceptibility. We further distinguish transient, inducible tolerance from stable, heritable resistance, and discuss how chronic subinhibitory exposure can drive their evolutionary interconversion. Extending beyond clinical pathogens, we highlight environmental microbiomes as major reservoirs of anti-AMP determinants with implications for horizontal transfer and One Health risk. Finally, we argue that AMP development and deployment must adopt a resistome-aware framework that integrates molecular mechanisms, evolutionary dynamics, and environmental context to preserve long-term therapeutic efficacy.

## 1. Introduction

Antimicrobial peptides (AMPs), also referred to as host defense peptides (HDPs), have long been regarded as agents that are largely resistant to the evolution of microbial resistance or at least less prone to resistance development compared with conventional antibiotics [1,2,3]. The rationale is that AMPs often have multi-target, membrane-disruptive modes of action, making it ostensibly difficult for bacteria to acquire a single genetic mutation to escape their lethal effect [4]. Indeed, some reviews have asserted that bacterial resistance to AMPs evolves only extremely slowly, posing “few concerns” for therapeutic use [5,6]. This conventional perspective has perpetuated the assumption that AMPs rarely promote the development of bacterial resistance [7,8].

However, an increasing body of evidence challenges this assumption. Under specific conditions such as prolonged low-dose exposure, growth within biofilms, or host-mimicking environments, bacteria are capable of developing stable tolerance or resistance to AMPs [6,9,10]. Pioneering experimental evolution studies have documented rapid resistance in the lab; for example, *Staphylococcus aureus* exposed to sub-inhibitory levels of the human cathelicidin LL-37 acquired significant resistance after only three serial passages [11]. With continuous AMP pressure over approximately 168 generations, *S. aureus* evolved stable, high-level resistance to LL-37. Similar induced resistance phenotypes have been observed in *Salmonella enterica* and *Clostridioides difficile* after repeated LL-37 exposure [12]. These findings underscore that resistance to AMPs is not universally inevitable, nor is it an isolated anomaly [13]. Rather, bacterial populations can acquire resistance under conditions of sustained selective pressure [13]. In fact, one analysis bluntly concluded that the development of AMP resistance is inevitable over time, given bacteria’s adaptive prowess [14].

The capacity of bacteria to resist AMPs is not the result of sporadic mutations or isolated adaptations, but instead reflects the operation of an integrated network of genetic and regulatory elements known as the anti-AMP resistome [15]. This resistome has evolved through prolonged co-selection alongside host immune peptides and enables bacteria to neutralize, expel, or tolerate AMPs under diverse conditions [1,6,16]. Contrary to the notion that AMP resistance is non-inducible or evolutionarily constrained, mounting evidence indicates that resistance mechanisms are both diverse and actively mobilized in response to AMP exposure [17,18]. However, unlike well-characterized resistomes associated with β-lactams or polymyxins, the anti-AMP resistome remains insufficiently defined and systematically underexplored [4].

This review aims to establish a comprehensive framework for understanding the anti-AMP resistome. We synthesize current knowledge on the molecular strategies by which bacteria evade or withstand AMP-mediated killing, examine ecological and environmental reservoirs of resistance traits, and assess their implications for the development and deployment of peptide therapeutics [19,20]. By delineating the architecture and dynamics of the anti-AMP resistome, we challenge the prevailing view that AMPs are inherently “resistance-proof” and advocate for a more anticipatory, resistome-informed approach to AMP discovery, optimization, and stewardship [21].

## 2. Host Innate AMPs as Primordial Selective Pressures

Long before humans considered using AMPs as drugs, bacteria have been constantly exposed to host-derived AMPs in nature [2,22]. Marine hosts add a distinctive dimension to this evolutionary landscape [23]. Fish skin and gill mucus, the epithelial surfaces of molluscs and annelids, and the hemolymph of crustaceans and horseshoe crabs are enriched in ribosomally synthesized eukaryotic HDPs [24,25]. Representative marine HDPs include fish piscidins and pleurocidin-like peptides, hepcidins and cathelicidins, mussel myticins and mytilins, shrimp penaeidins and crustins, oyster defensins and big defensins, lugworm arenicins, and horseshoe crab tachyplesins and polyphemusins [26,27,28]. These peptides are predominantly cationic and membrane-active, creating persistent selection on coastal microbiomes and aquaculture-associated communities [29,30]. Moreover, the skin, gastrointestinal tract, respiratory mucosa, and other barrier surfaces are continuously exposed to complex mixtures of endogenous AMPs [31]. For example, human epithelial tissues produce cathelicidins such as LL-37 and various defensins on land [1]. Similarly, nearly all multicellular organisms, including insects, mammals, and plants, synthesize their own AMPs as integral components of innate immunity [32]. These host-origin peptides impose a continuous selective pressure on the microbial communities that colonize those surfaces [33]. Commensals and opportunists that successfully inhabit skin or mucosal surfaces have, by necessity, evolved strategies to endure the onslaught of host AMPs [34]. For example, a recent study showed that a gut commensal (*Lactobacillus plantarum*) requires specific cell-wall modifications to resist host α-defensins, which is essential for its resilience during intestinal inflammation [15]. In fact, representative gut microbes across all major phyla show remarkable inherent resistance to high concentrations of host AMPs, underscoring how tolerance to AMPs is a prerequisite for long-term colonization in host environments [35].

**Figure 1:**
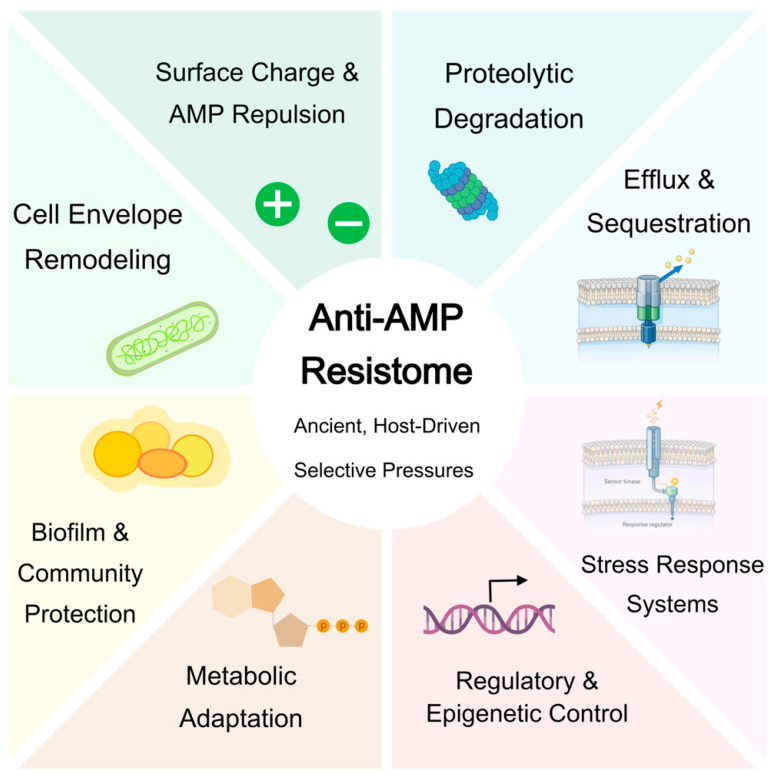
Host-driven selective pressures shape the anti-AMP resistome. Conceptual overview of the major bacterial strategies that collectively contribute to the anti-AMP resistome, including envelope remodeling, proteolytic degradation, efflux or sequestration, stress response activation, metabolic adaptation, and biofilm- or community-level protection. Created with MedPeer.

This ancient microbial arms race between host-derived AMPs and bacterial survival strategies has persisted for hundreds of millions of years [36,37]. Over evolutionary time, environmental and commensal bacteria have accumulated a broad arsenal of anti-AMP defenses, from cell envelope tweaks to regulated stress responses [38]. In essence, the “anti-AMP resistome” originates in nature’s own selective pressures. When we now introduce therapeutic AMPs into clinical use, we are pitting our designed molecules not against naïve bacteria, but against organisms that have been pre-conditioned by eons of exposure to similar host peptides [39]. The development timeline of a new AMP drug (from design to approval) is trivially short compared to the evolutionary timeline over which bacteria have optimized their AMP evasion strategies [40]. Thus, every therapeutic AMP is inherently entering a race against host-derived selective pressures that have already shaped a pre-existing resistome (Figure 1) [41,42]. Appreciating this dynamic reframes how we view AMPs: not as entirely novel agents to which bacteria have no defense, but as mimics of innate immune molecules for which nature’s “training” has already occurred [43,44]. In the following sections, we examine the composition of the anti-AMP resistome and its role as a latent facilitator of bacterial survival, which can be activated even prior to the introduction of synthetic AMPs.

## 3. Composition of the Anti-AMP Resistome

What exactly comprises the Anti-AMP Resistome? Broadly, it is the collection of genes, regulatory circuits, and phenotypic strategies that confer bacterial tolerance or resistance to killing by AMPs. As summarized in Table 1, the anti-AMP resistome encompasses both reversible tolerance states and stable resistance mechanisms that act in concert under peptide-mediated selective pressure. Drawing parallels to classical antibiotic resistomes, we can categorize anti-AMP defenses into distinct mechanistic modules [18]. These modules often work in concert to protect the cell, and many are induced or upregulated in the presence of AMPs [45]. Below, we outline six major components of the anti-AMP resistome (summarized in Figure 1 as a radial schematic), highlighting representative mechanisms and their roles.

### 3.1. Surface Charge Modulation: A Frontline Strategy for AMP Evasion

Cationic AMPs rely on electrostatic attraction to bind negatively charged bacterial surfaces [46]. These envelope-centered modifications represent the first structural layer of anti-AMP defense, acting primarily to reduce peptide binding, penetration, and local concentration at the cell surface. To counteract this, many bacteria actively modulate their surface charge, reducing peptide binding affinity and delaying membrane disruption [15]. This represents a conserved and highly inducible resistance mechanism, widespread across both Gram-negative and Gram-positive taxa [47].

The same electrostatic entry step governs many marine eukaryotic HDPs, including fish piscidins and pleurocidin-like peptides, as well as horseshoe crab tachyplesins, so surface charge tuning is expected to be a recurrent solution in coastal and aquaculture-associated bacteria that repeatedly encounter these cationic peptides [48,49,50,51].

In Gram-negative bacteria, lipopolysaccharide (LPS) remodeling is a key strategy [52]. Pathogens such as *Salmonella enterica* incorporate 4-amino-4-deoxy-L-arabinose (Ara4N) or phosphoethanolamine (PEtN) onto lipid A phosphate groups, neutralizing negative charges and diminishing AMP binding [53]. These modifications are catalyzed by enzymes like ArnT and EptA, which are tightly regulated by two-component systems such as PhoP/PhoQ and PmrA/PmrB. Functional loss of Ara4N modification sensitizes *Salmonella* to defensins and impairs virulence, underlining the protective role of surface charge alteration [54]. In marine Gram-negative pathogens, *Vibrio* species provide a particularly relevant model for anti-AMP adaptation [55]. *Vibrio* species occupy a unique ecological position as free-living marine bacteria, commensals, and opportunistic pathogens. This ecological plasticity allows anti-AMP traits selected in environmental reservoirs to be readily transferred into disease contexts, making *Vibrio* an effective conduit between environmental resistomes and aquaculture-associated infections [55,56]. Several *Vibrio* pathogens of fish and shellfish exhibit inducible lipid A remodeling, outer membrane charge modification, and enhanced biofilm formation in response to cationic HDPs, thereby reducing peptide binding and penetration [57].

In Gram-positive species, analogous strategies involve the modification of teichoic acids and membrane phospholipids [58]. The *dlt* operon mediates D-alanylation of wall teichoic acids, while *MprF* facilitates L-lysine addition to phosphatidylglycerol, both reducing net negative surface charge [59]. Deletion of either *dlt* or *mprF* significantly enhances bacterial susceptibility to AMPs such as human defensins and attenuates virulence in vivo, confirming their functional importance in resistance [58].

Beyond covalent modifications, some bacteria produce charged extracellular capsules composed of polyanionic polysaccharides that serve as decoys to sequester AMPs [60]. These structures act as electrostatic buffers, binding AMPs in the extracellular space and preventing peptide access to the membrane [61]. For instance, *Streptococcus* strains with hyaluronic acid capsules demonstrate higher AMP tolerance than their acapsular counterparts.

Importantly, surface charge modulation is not constitutive but highly responsive. Upon sensing AMP exposure, bacteria rapidly upregulate genes such as *arnT*, *eptA*, *dlt*, and *mprF* through specific signaling pathways (see Section 3.5). This dynamic, inducible system enables bacteria to transiently cloak themselves from AMP action, providing time to activate secondary resistance modules [61].

In sum, by tuning the electrostatic landscape of the cell envelope, bacteria deploy a robust, layered defense that directly impairs AMP binding and membrane interaction. This strategy forms a foundational component of the anti-AMP resistome and is a primary target for therapeutic peptide redesign [62].

### 3.2. Reinforcing Envelope Barriers: Passive and Active Peptide Exclusion

Beyond surface charge modulation, bacteria deploy a second critical line of defense by strengthening the structural and functional integrity of the outer membrane or cell wall, thereby reducing AMP access to vulnerable cytoplasmic targets [63]. This form of barrier fortification includes passive exclusion, achieved by decreasing membrane permeability, as well as active defense strategies such as the activation of efflux systems and the production of molecular decoys [64].

These exclusion mechanisms are particularly relevant in marine settings where pathogens of fish and shellfish experience sustained HDP exposure at mucosal surfaces and within surface-associated biofilms on nets, tanks, and biofilters, thereby reducing the fraction of peptide that reaches the cytoplasmic membrane [57,65].

In Gram-negative bacteria, outer membrane porins represent a key entry route for small peptides [66]. Selective downregulation or structural alteration of porins can significantly impede AMP uptake [67]. Additionally, certain peptide transporters that originally evolved to facilitate nutrient uptake can unintentionally mediate the internalization of AMPs [68]. The SapABC transporter system exemplifies this dual role: while facilitating peptide uptake, it can also target AMPs for intracellular degradation. Mutants lacking Sap function exhibit heightened AMP sensitivity, suggesting a dual role in both peptide import and detoxification [69].

Gram-positive bacteria, lacking an outer membrane, rely more heavily on cell wall remodeling and active efflux [70]. ABC transporters such as VraFG in *Staphylococcus aureus* and BceAB in *Bacillus subtilis* can export AMPs directly or contribute to signal transduction cascades that upregulate broader resistance responses [71]. These transporters are often co-regulated with sensor systems (e.g., GraRS), enabling rapid peptide-triggered activation of resistome components [66].

Efflux mechanisms with broader substrate profiles also contribute to anti-AMP defense. Resistance–nodulation–division family efflux pumps such as MexAB-OprM in *Pseudomonas aeruginosa*, a species well known for its multidrug resistance, have been shown to reduce intracellular concentrations of representative AMPs [72,73]. While these pumps vary in efficiency against different peptides, their broad specificity enhances survival under peptide-rich conditions and links AMP resistance to multidrug tolerance phenotypes [74].

Envelope fortification is further supported by vesicle-based sequestration. Many Gram-negatives respond to envelope stress by hyperproducing outer membrane vesicles (OMVs), which act as molecular decoys by absorbing and neutralizing AMPs before they can reach the bacterial surface [43]. These vesicles, rich in LPS and membrane proteins, bind cationic peptides with high affinity and reduce their effective concentration near the cell envelope [75].

In parallel, Gram-positive bacteria reinforce their peptidoglycan (PG) matrix. Increased cross-linking density, altered glycan chain length, and the incorporation of protective modifications can all slow AMP diffusion and entrap peptides within the cell wall [76]. For instance, metabolic changes that alter nitrogen flux, such as glutamine synthetase overexpression, have been linked to increased PG thickness and AMP resistance [43]. This suggests that envelope reinforcement is not purely structural, but metabolically plastic and environmentally responsive [75].

Collectively, these mechanisms constitute a broad-spectrum resistance module that limits AMP access via physical insulation, active export, and peptide sequestration. Their widespread presence across phylogenetically diverse bacteria highlights their evolutionary importance, while their inducibility under peptide stress reflects a dynamic and resource-efficient deployment of the resistome [77].

### 3.3. Extracellular Proteolysis of AMPs

A powerful mechanism by which bacteria neutralize AMPs is the extracellular secretion of proteases that cleave these peptides into inactive fragments [78]. This strategy eliminates the antimicrobial activity before AMPs can engage their targets and represents a frontline mode of resistance among both pathogenic and environmental bacteria [79].

Marine HDPs are also vulnerable to this route of inactivation, particularly linear amphipathic peptides such as fish piscidins and cathelicidins, meaning that protease-rich coastal habitats and aquaculture systems can attenuate both endogenous host protection and peptide-based interventions [80,81].

Numerous bacterial pathogens secrete broad-spectrum proteases capable of degrading host-derived AMPs such as LL-37 and defensins [82]. *Pseudomonas aeruginosa* produces LasB elastase, which cleaves LL-37 at specific sites, rapidly abolishing its bactericidal activity. *Staphylococcus aureus* secretes aureolysin and V8 protease, which similarly dismantle human AMPs [83]. In *Streptococcus pyogenes*, the cysteine protease SpeB targets a wide range of host peptides. These enzymes are often active against multiple AMP classes, underscoring their broad substrate specificity and functional plasticity [84].

Proteolytic inactivation of AMPs substantially enhances bacterial survival. In ex vivo human wound fluid models, the addition of purified elastase from *P. aeruginosa* results in rapid depletion of LL-37, accompanied by a marked increase in bacterial viability [85]. Conversely, pharmacological inhibition of this protease restores the killing activity of LL-37, directly implicating AMP degradation as the survival-enabling factor. Protease expression is frequently upregulated in response to infection, inflammation, or biofilm formation, all of which are conditions associated with elevated AMP exposure [86,87].

Beyond generalist proteases, some bacteria possess specialized systems for targeted AMP degradation. *Salmonella* utilizes the SapABC transporter to import AMPs into the periplasm, where peptidases subsequently degrade them, effectively forming a coordinated capture-and-destroy pathway [88]. *S. aureus* secretes staphylokinase, which co-opts host plasminogen into an active protease capable of cleaving defensins, representing a form of host-directed AMP neutralization. Regulation of these proteases is often controlled by quorum sensing systems, such as LasR in *P. aeruginosa*, ensuring efficient expression during high-density states or within structured communities [89,90,91].

This proteolytic strategy offers several advantages. It can render bacteria fully resistant to specific AMPs, regardless of peptide concentration, and often carries minimal fitness costs in host-associated environments where these enzymes have dual roles in nutrient acquisition or virulence. Many protease genes are stably maintained in core genomes or readily disseminated via horizontal gene transfer. Collectively, AMP proteolysis is a pervasive, evolutionarily conserved, and mechanistically diverse arm of the anti-AMP resistome [92].

### 3.4. Extracellular Matrix and Biofilm-Mediated Buffering

Bacteria frequently organize into biofilms, which are structured communities encased in a self-produced extracellular matrix composed of polysaccharides, proteins, and extracellular DNA. This matrix provides a protective microenvironment that significantly attenuates the activity of AMPs. Biofilm-associated tolerance to AMPs involves both physical shielding and physiological adaptations [93].

This buffering is highly relevant to marine environments and aquaculture, where biofilms develop on cages, nets, pipelines, and biofilters and coexist with continuous inputs of endogenous fish and shellfish HDPs, thereby prolonging subinhibitory exposure and intensifying selection for tolerant phenotypes [94,95].

One of the primary mechanisms is diffusion restriction. The dense extracellular polymeric substance (EPS) network acts as a barrier that impedes the penetration of AMPs into the biofilm interior. Positively charged peptides such as LL-37 may bind to negatively charged matrix components, including extracellular DNA and acidic polysaccharides, thereby reducing their mobility and neutralizing their activity. As a result, AMPs often fail to reach lethal concentrations at deeper biofilm layers. This spatial sequestration contributes to the remarkable tolerance observed in biofilm-grown bacteria compared to their planktonic counterparts [96].

The matrix also functions as a molecular sink that absorbs and neutralizes AMPs. Extracellular DNA can chelate cationic peptides, while matrix-associated anionic polymers such as alginate in *Pseudomonas aeruginosa* or poly-γ-glutamate in *Staphylococcus aureus* efficiently bind and inactivate AMPs [91]. Even structural proteins and embedded outer membrane vesicles contribute to AMP interception. These interactions lower the effective AMP concentration at the cell surface, producing an apparent resistance that would not be observed in matrix-free conditions [92].

Biofilm-specific physiological changes further enhance AMP tolerance. Reduced metabolic activity, altered membrane potential, and the presence of persister cells all contribute to decreased susceptibility [97]. In addition, stress response pathways activated during biofilm formation can cross-protect against peptide-mediated damage. For instance, the extracytoplasmic function sigma factor SigW in *Bacillus subtilis* regulates envelope stress response genes that enhance resistance to bacteriocins such as nisin [98].

Importantly, sub-inhibitory exposure to cationic peptides can reinforce matrix production. In *Pseudomonas*, polymyxin B has been widely used as a mechanistic comparator to demonstrate inducible alginate upregulation, illustrating how peptide stress can feed back into biofilm fortification [99]. This response amplifies the protective barrier and exemplifies an inducible defense strategy, where AMP sensing promotes structural fortification [100].

These mechanisms establish the biofilm matrix as both a passive shield and an active participant in AMP resistance [101]. Although this tolerance is typically reversible upon dispersal, its clinical implications are profound. Biofilm formation underlies chronic infections in settings such as cystic fibrosis lungs, chronic wounds, and implanted medical devices [102]. Overcoming matrix-mediated AMP resistance remains a key challenge in the development of effective peptide-based therapies. Together, envelope remodeling and matrix-mediated buffering define a physical defense layer that constrains AMP access before intracellular targets are engaged.

### 3.5. Quorum Sensing and Two Component Regulatory Systems

Bacteria have evolved dynamic regulatory architectures that actively detect and respond to AMPs. Among the most studied are two-component systems (TCSs), which comprise a membrane-bound sensor kinase and a cytoplasmic response regulator [103]. Rather than functioning as independent resistance mechanisms, two-component systems and quorum sensing circuits operate as the central regulatory layer of the anti-AMP resistome, coordinating the deployment of structural defenses in response to peptide exposure. These systems detect environmental cues such as AMPs and coordinate the induction of resistance effectors. Quorum sensing pathways, which monitor cell population density, can also shape the collective response to peptide threats by modulating community behaviors such as biofilm formation and protease secretion [104].

Comparable peptide-sensing circuits likely operate in many marine and aquaculture-associated bacteria, for which endogenous HDPs such as piscidins, hepcidins, penaeidins, and bivalve defensins represent plausible ecological cues that coordinate envelope remodeling, efflux, and biofilm programs [95,105,106].

In *Staphylococcus aureus* and *S. epidermidis*, the GraRS system (also known as Aps) serves as a central AMP-responsive module. Upon sensing cationic peptides such as LL-37 or defensins, the GraS kinase phosphorylates the response regulator GraR, which activates multiple resistance genes [107]. These include *mprF* for lysinylation of phospholipids, the *dltABCD* operon for teichoic acid modification, and the *vraFG* efflux system. These modifications decrease peptide binding affinity and enhance export of toxic compounds [108]. Notably, clinical isolates of *S. aureus* often carry mutations in *graS* that constitutively activate this system, suggesting in vivo selection by host peptides. In *S. epidermidis*, the Aps system responds to a broader spectrum of peptides, reflecting its adaptation to skin colonization under polymicrobial conditions [109].

Gram-negative bacteria rely heavily on the PhoPQ and PmrAB systems to sense AMP-rich environments. PhoQ detects low magnesium, acidic pH, and cationic peptides, triggering PhoP-mediated activation of LPS remodeling genes such as *arnBCADTEF* and *eptA* [110]. These modifications add positively charged residues to lipid A, reducing peptide binding. Disruption of *mgrB*, a negative regulator of PhoPQ, commonly yields constitutive activation and is a well-established route to resistance against polymyxins such as colistin, which are non-ribosomally synthesized bacterial lipopeptide antibiotics. Notably, the same lipid A remodeling program can also reduce susceptibility to HDPs, underscoring that AMP-sensing circuits integrate ecological and clinical pressures through shared envelope adaptations [111].

Additional AMP-responsive TCSs include ParRS and ColRS in *Pseudomonas aeruginosa*, which control efflux pump expression and outer membrane alterations. Other pathogens such as *Campylobacter jejuni* and *Neisseria meningitidis* utilize regulatory systems that adjust capsule or LOS composition in response to AMP exposure [3,112]. Global stress regulators like σ^B in *Listeria monocytogenes* and *Staphylococcus* species also enhance tolerance indirectly by inducing cell wall thickening and repair systems [113,114].

Quorum sensing influences AMP resistance at the population level. In *P. aeruginosa*, the Las and Rhl systems coordinate the expression of proteases such as LasB elastase and alkaline protease, which degrade peptides extracellularly [115]. QS also promotes biofilm formation and exopolysaccharide production, providing physical barriers to AMP penetration [116]. Furthermore, there is evidence of feedback loops wherein sublethal AMP exposure stimulates QS-regulated matrix production, reinforcing community-level resistance [116].

These regulatory systems constitute the command network of the anti-AMP resistome. Rather than relying on constitutive expression, bacteria utilize environmental sensing to deploy resistance modules precisely when needed [117]. This not only minimizes fitness cost but also enables rapid adaptation [118]. From a therapeutic perspective, targeting regulatory hubs such as GraR or PhoP may disable multiple resistance pathways simultaneously [119]. The existence of such specialized sensing circuits provides compelling evidence that AMPs exert substantial selective pressure in natural and clinical environments, contrary to the notion that resistance to host peptides is evolutionarily rare [120].

### 3.6. Stress Responses and Cross-Protection Phenomena

**Figure 2:**
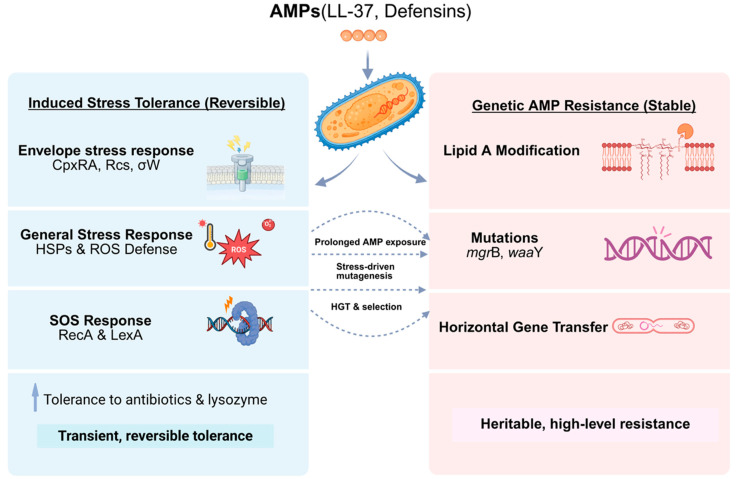
Induced tolerance and genetic resistance as two tiers of the anti-AMP resistome. Conceptual overview of the major bacterial strategies that collectively contribute to the anti-AMP resistome, including envelope remodeling, proteolytic degradation, efflux or sequestration, stress response activation, metabolic adaptation, and biofilm- or community-level protection. Created with MedPeer.

AMPs frequently act as environmental stressors that activate broad-spectrum defense responses in bacteria (Figure 2) [121]. Stress response pathways represent an integrative layer of the anti-AMP resistome, linking short-term physiological survival to longer-term adaptive and evolutionary trajectories. These stress responses are often not AMP-specific but nonetheless contribute to heightened peptide tolerance and can incidentally enhance survival against unrelated antibiotics or environmental insults. As such, they form a diffuse yet critical component of the anti-AMP resistome [122].

In marine and aquaculture microbiomes, these general stress programs are repeatedly engaged by fluctuating salinity, oxidative pulses, and co-exposure to disinfectants and trace metals, which may amplify cross-protection and complicate peptide-based disease control. Envelope stress responses are a central part of this module. In Gram-negative bacteria, systems such as CpxRA and Rcs detect cell envelope perturbations and trigger protective remodeling, while Gram-positive species utilize regulators like σ^W to upregulate cell wall fortification and efflux pathways [123]. These changes increase resilience not only to AMPs but also to surfactants, lysozyme, and certain antibiotics. The overlap in resistance phenotypes arises because many of the induced genes, such as efflux pumps and lipid-modifying enzymes, have broad substrate specificity. Thus, exposure to host-derived AMPs can precondition bacteria to withstand chemically distinct antimicrobials [124].

In cases where AMPs penetrate the membrane and reach intracellular targets, DNA damage and replication stress can ensue [125]. This activates the SOS response, a global regulatory system centered on RecA-mediated cleavage of the LexA repressor. RecA is increasingly recognized as a core determinant of AMP resistance [126]. Its functions in DNA repair, recombination, prophage induction, and horizontal gene transfer collectively increase bacterial survival under AMP challenge. In *Acinetobacter baumannii*, polymyxin-resistant strains, including those selected by colistin exposure, can show RecA dependence for maintaining viability and promoting biofilm formation [127,128]. These processes contribute not only to immediate survival but also to long-term resistance evolution.

An important dimension of stress-induced resistance involves cross-protection between innate immunity and clinical antibiotics [128]. The best-characterized example is the plasmid-borne *mcr-1* gene, which confers resistance to colistin, a non-ribosomally synthesized polymyxin antibiotic, by phosphoethanolamine modification of lipid A. This modification can also reduce susceptibility to HDPs such as LL-37, thereby creating clinically relevant cross-protection between peptide antibiotics and innate immune peptides. Conversely, experimental evolution of bacteria under AMP pressure often results in mutations that also confer antibiotic resistance, as observed with *mgrB* and *waaY* in *Salmonella* [129]. This reciprocal selection highlights the interconnected nature of peptide-induced and drug-induced resistance, with implications for both therapeutic use and environmental exposure [130].

General stress hardening contributes further to transient resistance states. Sublethal AMP exposure can trigger ion influx and cytoplasmic stress pathways, resulting in the upregulation of heat shock proteins, chaperones, and oxidative stress defenses [131]. These factors may transiently reduce susceptibility to antibiotics, akin to adaptive resistance [132]. For instance, an AMP challenge can prime bacteria to survive a subsequent antibiotic insult more effectively, even in the absence of genetic changes [133].

These stress responses reflect the integrative nature of bacterial survival strategies [134]. AMP exposure perturbs regulatory networks that extend well beyond direct resistance mechanisms, often reinforcing unrelated tolerance traits [135]. From a One Health perspective, the implications are significant: bacteria exposed to AMPs or biocides in agricultural or environmental settings may carry cross-protected phenotypes into clinical contexts [136]. This underlines the importance of considering AMP-induced stress responses as both direct and indirect drivers of antimicrobial resistance evolution [137]. Viewed collectively, these stress-associated responses do not constitute redundant defenses but rather amplify and stabilize upstream structural and regulatory mechanisms, thereby accelerating the transition from inducible tolerance to heritable resistance.

To provide a structured overview of the molecular strategies that collectively constitute the anti-AMP resistome, the major resistance mechanisms described throughout this Review are summarized in Table 2. These mechanisms span surface charge modulation, membrane remodeling, efflux, extracellular proteolysis, biofilm-mediated buffering, and stress-responsive regulatory circuits, and are conserved across diverse bacterial taxa. Importantly, the table highlights that many of these strategies operate against eukaryotic HDPs from both terrestrial and marine organisms, including fish, invertebrate, and annelid-derived HDPs, underscoring the evolutionary breadth and ecological relevance of the anti-AMP resistome.

**Table 2:** Major anti-AMP resistance mechanisms and representative examples.

Resistance Mechanism	Molecular Basis	Representative AMPs/HDPs	Representative Bacteria	Key References
Surface charge modulation	*dlt* operon–mediated D-alanylation of teichoic acids; *mprF*-mediated lysyl-phosphatidylglycerol synthesis; lipid A modification via *PhoPQ/PmrAB*	LL-37; human defensins; fish piscidins; lugworm arenicins	*Staphylococcus aureus*; *Salmonella enterica*; *Vibrio* spp.	[138,139,140]
Outer membrane remodeling	Addition of Ara4N or phosphoethanolamine to lipid A; altered acylation patterns	LL-37; tachyplesins; pleurocidin-like peptides	*Salmonella*; *Klebsiella*; *Vibrio* spp.	[141,142]
Efflux-mediated peptide removal	RND, MFS, and ABC transporters; GraRS-regulated VraFG	Cationic amphipathic HDPs	*S. aureus*; *Pseudomonas aeruginosa*; *Vibrio* spp.	[143,144]
Extracellular proteolysis	Secreted metalloproteases, serine and cysteine proteases degrading linear AMPs	LL-37; piscidins; cathelicidins	*Pseudomonas*; *Vibrio*; *Streptococcus*	[145,146]
Capsule and matrix sequestration	Anionic polysaccharides; extracellular DNA binding cationic peptides	LL-37; marine HDPs	*Vibrio* spp.; *Pseudomonas* spp.	[147,148]
Biofilm-mediated buffering	EPS-mediated diffusion limitation; maintenance of subinhibitory exposure	Broad-spectrum HDPs	*Vibrio* spp.; aquaculture-associated consortia	[149,150]
Two-component regulatory systems	AMP sensing via GraRS, PhoPQ, PmrAB, ParRS	Broad class of cationic HDPs	*S. aureus*; *Salmonella*; *Vibrio* spp.	[151,152]
Stress response cross-protection	SOS response; oxidative stress pathways; reduced growth states	LL-37; piscidins	Multiple taxa	[153,154]

## 4. Environmental Reservoirs of a Broad-Spectrum Anti-AMP Gene Pool

While AMP resistance in pathogens is often viewed through the lens of host-associated evolution, evidence increasingly points to the environment as a vast and ancient reservoir of anti-AMP mechanisms [155]. Diverse microbial communities in soil, water, and animal waste are constantly exposed to natural antimicrobial pressures and have evolved broad-spectrum defenses accordingly [156,157,158]. These environmental resistome elements can serve as a pre-adapted gene pool for pathogens, facilitating rapid resistance acquisition against novel therapeutic AMPs.

Environmental microbes are routinely challenged by AMPs originating from plants, animals, insects, and other microbes [159]. For instance, aquatic bacteria encounter peptides from fish mucus and insect larvae, while soil microbes face plant defensins and insect-derived toxins [160]. In marine and coastal systems, selection is reinforced by dense eukaryotic HDPs landscapes at animal surfaces and in aquaculture settings, where piscidins, hepcidins, penaeidins, myticins, and big defensins can act as continual filters on surrounding microbiota and enrich broadly protective envelope and biofilm traits [161,162]. In aquaculture systems, *Vibrio* spp. are continuously exposed to HDPs at mucosal surfaces and within surface-associated biofilms on nets, tanks, and biofilters. These conditions create sustained sublethal peptide exposure that favors the selection of tolerance and resistance traits in *Vibrio* populations, with direct implications for disease persistence and treatment failure in marine farming [163,164]. In agricultural runoff and sewage, microbes are exposed to AMPs derived from animal immune systems. These selective forces enrich for generalized resistance traits, forming a “generalist” resistome capable of countering diverse peptides [160,165]. Comparative metagenomic studies show that the soil microbiome harbors a larger and more diverse collection of AMP resistance genes than the human gut microbiome, reflecting the intensity and variety of environmental selective pressures.

Marine AMPs exert ecological effects that extend beyond direct pathogen inhibition [166]. In coastal waters, host-associated niches, and aquaculture systems, chronic exposure to eukaryotic HDPs acts as a persistent selective force shaping microbial community composition and function [148,167]. Rather than uniformly suppressing microbial growth, marine HDPs preferentially filter sensitive taxa while enriching populations with intrinsic tolerance traits, thereby restructuring microbiota toward peptide-adapted assemblages [167]. Biofilm-associated communities are particularly affected, as extracellular matrices concentrate cationic peptides and maintain subinhibitory exposure over extended periods [147,148,149]. Under these conditions, HDPs can shift community metabolism, promote slow-growing or stress-tolerant phenotypes, and increase the relative abundance of taxa equipped with envelope-modifying enzymes, efflux systems, and proteolytic capacities [147,149]. Such microbiota-level shifts are consistent with observations from aquaculture biofilters, net-associated biofilms, and host mucosal microbiomes, where community restructuring can couple to functional changes relevant to water quality and pathogen persistence [148,168]. Importantly, these microbiota-level effects imply that marine HDPs influence not only individual resistance phenotypes but also the collective properties of microbial communities. By selecting for peptide-tolerant consortia, HDPs can indirectly facilitate the emergence and stabilization of anti-AMP resistomes at the ecosystem scale, with implications for disease dynamics, environmental resilience, and long-term sustainability of peptide-based interventions in marine systems [148,167].

Notably, the environmental and clinical resistomes are not isolated systems. Sequence-based analyses have revealed homologous AMP resistance genes in both settings, including genes with high identity to those in *Klebsiella pneumoniae* and *Pseudomonas aeruginosa*. This overlap implies that environmental bacteria act as evolutionary incubators where resistance traits emerge, some of which later transition into human pathogens through horizontal gene transfer or via opportunistic species that bridge both niches [169,170,171].

Mobile genetic elements further facilitate this transfer. Many AMP resistance genes reside on plasmids, transposons, or integrons, enabling their dissemination across microbial populations [172]. The *mcr-1* gene, encoding a phosphoethanolamine transferase conferring colistin resistance, exemplifies this process [173]. Likely originating in environmental *E. coli* from pig farms, *mcr-1* has now disseminated globally into clinical pathogens. Its activity not only confers resistance to colistin, a clinically used non-ribosomally synthesized cationic lipopeptide antibiotic, but also reduces bacterial susceptibility to HDPs such as LL-37. Metagenomic surveys have also detected co-localization of AMP resistance genes with classical antibiotic resistance genes (ARGs), suggesting the possibility of co-selection, where antibiotic use inadvertently promotes AMP resistance and vice versa [174].

Opportunistic pathogens further act as “mixing vessels” that shuttle resistance traits between environmental and clinical contexts [175]. Species such as *Pseudomonas aeruginosa*, *Acinetobacter baumannii*, and *Stenotrophomonas maltophilia* are commonly found in soil and water yet frequently cause human infections [176]. These organisms often possess intrinsic resistance systems shaped by environmental survival, including preexisting tolerance to host AMPs. For example, *P. aeruginosa* isolates from natural waters exhibit notable AMP resistance despite no prior exposure to human hosts, likely due to adaptation against protozoan predation and environmental peptides. Such pre-adaptation undermines the assumption that therapeutic AMPs will face naïve targets [177].

The cumulative evidence suggests that the environment serves as both a historical crucible and an ongoing source of anti-AMP resistance [178]. From a One Health perspective, this ecological dimension is critical [179]. The use of peptide-based antimicrobials must account for environmental reservoirs that already harbor similar molecules and corresponding resistance determinants. In several cases, environmental isolates have exhibited non-susceptibility to candidate AMPs in development, equipped with robust efflux systems or proteolytic enzymes [180]. Ignoring this background may lead to underestimating the speed and scale at which resistance can spread [180].

In summary, environmental microbiomes maintain a deep and dynamic reservoir of anti-AMP genes shaped by natural selection. These elements can enter clinical settings through genetic transfer or via inherently resistant pathogens, posing a latent but significant threat to AMP therapeutics. Future drug development, deployment, and stewardship must integrate environmental surveillance to anticipate and mitigate these cross-ecosystem resistance flows.

## 5. Induced Tolerance Versus Permanent Resistance: Dynamics of AMP Resistance Development

**Figure 3:**
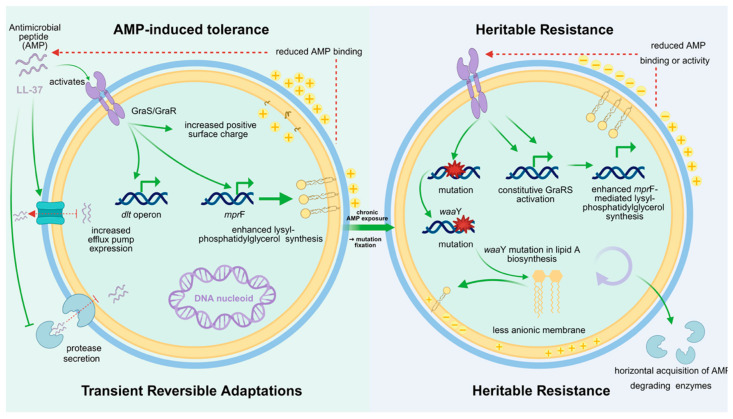
From AMP-induced tolerance to heritable resistance. Illustration of the continuum from transient, inducible tolerance to stable, heritable resistance under increasing duration and intensity of AMP exposure. Created with BioRender.

Bacterial resistance to AMPs is not a binary state but a continuum ranging from transient, inducible tolerance to stable, heritable resistance (Figure 3) [181]. Understanding this distinction is essential, as the two forms differ in their underlying mechanisms, evolutionary dynamics, and clinical consequences [182]. Early studies that claimed AMPs were unlikely to elicit resistance often examined only short-term exposures, capturing transient tolerance rather than true genetic adaptation.

Induced tolerance arises when AMP exposure triggers rapid physiological adjustments that temporarily reduce susceptibility [183]. These responses are typically mediated by regulatory circuits such as two-component systems and quorum sensing pathways [184]. Upon encountering sublethal concentrations of an AMP, bacteria may transiently increase cell wall thickness, upregulate efflux pumps, or enhance protease secretion. Such modifications reduce AMP binding or accelerate peptide clearance but revert once the stress subsides [185]. In *Staphylococcus aureus*, for instance, LL-37 exposure induces upregulation of the *dlt* operon and *mprF*, increasing surface positive charge and reducing peptide binding [185,186]. These changes dissipate when the peptide is removed, restoring baseline sensitivity. This form of tolerance, sometimes described as an adaptive response, is reversible and energetically costly, arising within minutes and disappearing after a few generations [187]. It explains why many laboratory assays, limited to single exposure cycles, fail to detect apparent resistance: cells survive through reversible adaptation rather than stable mutation [188].

Heritable resistance emerges through prolonged or repeated AMP exposure, which selects for mutations or gene acquisitions that fix resistance traits in the genome [189]. Stable resistance can result from point mutations in regulatory genes that render defense systems constitutively active or from structural alterations that diminish AMP binding [190]. In *S. aureus*, mutations in *graS* permanently activate the GraRS regulatory system, while changes in *mprF* increase lysyl-phosphatidylglycerol synthesis, creating a persistently more cationic membrane [186]. In *Salmonella*, long-term LL-37 exposure leads to mutations in lipid A biosynthesis genes such as *waaY*, yielding a permanently less anionic outer membrane [191]. Heritable resistance can also arise from horizontal gene transfer, exemplified by plasmid-encoded AMP-degrading enzymes or surface modification genes. In some *Pseudomonas* isolates, high-level AMP resistance required multiple mutations across independent loci, indicating that full resistance may be polygenic and evolve incrementally under sustained pressure [192].

The conceptual boundary between tolerance and resistance is not always clear-cut. Persistent activation of inducible pathways can facilitate the fixation of genetic mutations that stabilize the tolerant state [193]. For instance, continuous activation of the GraRS system during chronic infection can favor *graS* mutants that maintain constitutive expression of AMP resistance genes, effectively transforming a regulatory response into a heritable trait [194]. This progression from inducible tolerance to genetically encoded resistance highlights how chronic exposure at sublethal concentrations, whether occurring within host environments or external ecological reservoirs, can accelerate the evolution of stable resistance to AMPs [195].

A major limitation in AMP research has been the short temporal scope of experimental designs [196]. Many studies assess resistance development within a single growth cycle, overlooking cumulative adaptive changes that manifest only after serial passaging. Recent long-term evolution experiments demonstrate that AMP resistance can indeed emerge over extended timescales, challenging the long-held view that AMPs are “resistance-proof”. Furthermore, transcriptomic and proteomic analyses reveal that AMP-induced tolerance involves broad regulatory shifts beyond canonical defense pathways, including genes linked to metabolism and DNA repair [197]. These findings suggest that AMP exposure engages a complex, multilayered response network that we are only beginning to understand [198].

Recognizing the difference between transient and permanent resistance has direct therapeutic implications. Induced tolerance may lead to temporary treatment failure, requiring higher AMP concentrations or combination therapies, whereas heritable resistance represents a durable loss of efficacy and a potential for horizontal dissemination [199,200]. Effective AMP stewardship must therefore distinguish between adaptive tolerance that can be managed pharmacologically and permanent resistance that demands novel peptide design or adjunctive inhibitors targeting resistance machinery [201].

## 6. Implications for Therapeutic AMP Design: Pre-Existing Defenses and Avoiding the Obvious Pitfalls

**Figure 4:**
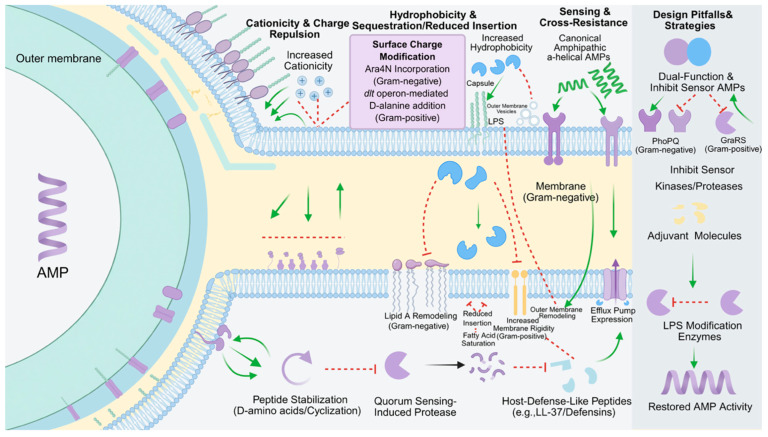
Interplay between the anti-AMP resistome and therapeutic AMP design. Schematic showing how major resistance mechanisms intersect with common AMP design principles and highlighting resistance-aware strategies to improve therapeutic performance. Created with BioRender.

An improved understanding of the anti-AMP resistome also provides a rational foundation for AMP design. Rather than optimizing peptides solely for maximal potency, effective translational strategies must anticipate and counteract bacterial resistance pathways. Based on the resistance mechanisms outlined above, Table 3 summarizes key design principles that link specific resistome pressures to concrete engineering strategies, with representative examples relevant to both clinical and marine applications. The breadth of resistance mechanisms identified in environmental and clinical isolates underscores a key challenge for AMP development: most candidate peptides are engineered with potency in mind but not with the resistome in sight (Figure 4) [202]. Strategies that optimize charge, hydrophobicity, or protease resistance often clash directly with well-established bacterial defenses [203]. Designing next-generation AMPs thus requires a shift from offense-centric optimization to resistance-aware engineering [204].

One frequent optimization involves enhancing peptide cationicity to improve binding to negatively charged bacterial membranes [205]. Yet many pathogens, including *E. coli* and *S. aureus*, possess inducible systems that increase their own surface charge through incorporation of molecules such as Ara4N or D-alanine. The higher the peptide’s positive charge, the stronger the electrostatic repulsion from such modified surfaces [206]. For example, in *S. aureus*, *dlt* operon activation effectively neutralizes even highly cationic peptides [207]. Excessive charge enhancement may yield diminishing returns, as it intensifies the selection for charge-based repulsion mechanisms [208].

Hydrophobicity is similarly a double-edged sword. Increasing hydrophobic content improves membrane disruption but also facilitates sequestration by bacterial capsules, lipopolysaccharides, or outer membrane vesicles [209]. Gram-negative bacteria can remodel lipid A to reduce AMP insertion, while Gram-positives increase membrane rigidity by altering fatty acid saturation [210]. In extreme cases, hyper-hydrophobic peptides may be trapped before reaching their target, particularly in species with rich outer envelope architecture [211].

To improve peptide half-life, stability against proteases is often pursued through D-amino acid substitution or cyclization. However, AMP exposure itself can trigger bacterial upregulation of proteases via quorum sensing, increasing extracellular enzymatic load [212]. This escalation may erode the protective advantage of such modifications [213]. Moreover, bacteria may deploy orthogonal inactivation strategies such as binding proteins or peptide modifications, expanding the battleground beyond mere cleavage resistance [214].

Canonical design templates, such as amphipathic α-helices, also present pitfalls. These structural motifs are recognized by bacterial sensory systems like PhoPQ and GraRS, triggering well-orchestrated countermeasures, including outer membrane remodeling and efflux pump activation [215]. In contrast, noncanonical structures that evade recognition or act intracellularly may avoid immediate detection and delay resistance induction. This highlights the need to anticipate not only how a peptide kills but how the bacterium perceives and responds to it [216].

Cross-resistance with HDPs further complicates design. Therapeutic AMPs often mimic or derive from innate molecules like LL-37 or defensins [217]. Pathogens that evolved to resist host immunity via LPS modification, surface remodeling, or proteolytic degradation are often preadapted to neutralize AMP drugs with similar physicochemical profiles [218]. Structural divergence from human AMPs may help circumvent this issue, though care must be taken to maintain safety and host compatibility [219].

Incorporating resistome-awareness into design requires multifaceted strategies. One avenue involves dual-function peptides that combine bactericidal activity with suppression of resistance mechanisms, such as simultaneously targeting membranes and inhibiting sensor kinases or proteases [220]. Another promising route is combination therapy: pairing AMPs with small-molecule adjuvants that block key resistance pathways, like LPS modification enzymes, may restore or enhance peptide efficacy [221].

Evaluation pipelines must evolve accordingly. Screening peptides solely against wild-type lab strains risks overestimating efficacy [221]. Instead, candidates should be challenged against strains engineered to overexpress resistance determinants, such as LPS-modified *E. coli* or high *dlt S. aureus*. Similarly, computational modeling of peptide-membrane interactions should incorporate modified bacterial envelopes to better predict clinical performance [222].

In sum, the design of AMPs must move beyond pharmacological optimization toward dynamic co-evolutionary thinking. An effective AMP is not just one that kills efficiently, but one that anticipates, neutralizes, or bypasses bacterial countermeasures. Understanding the logic and modular organization of the anti-AMP resistome does not hinder rational design; instead, it provides the conceptual key for advancing AMP development [223,224,225].

**Table 3:** Rational design strategies for AMPs informed by anti-AMP resistance mechanisms.

Resistance Pressure	Design Principle	Strategy	Representative Examples	Translational Relevance	Key References
Surface charge repulsion	Avoid extreme cationicity	Moderate net charge; spatial charge distribution	Engineered piscidin analogs	Reduced induction of envelope remodeling	[226,227,228]
Proteolytic degradation	Increase protease resilience	D-amino acid substitution; backbone cyclization	Cyclic defensin analogs	Enhanced stability in protease-rich aquaculture systems	[229,230]
Efflux-mediated clearance	Minimize pump recognition	Non-canonical residues; altered amphipathicity	Peptidomimetics	Sustained intracellular activity	[231,232,233]
Biofilm sequestration	Penetrate or disrupt matrix	Biofilm-active motifs; EPS-binding domains	Hybrid AMPs	Improved efficacy against surface-associated infections	[149,234,235]
Rapid sensor activation	Evade AMP-sensing TCS	Trojan-horse uptake; intracellular targets	Proline-rich peptides	Delayed resistome activation	[236,237]
Cross-resistance with host immunity	Structural divergence from endogenous HDPs	Non-natural scaffolds	Synthetic AMP mimics	Reduced immune cross-resistance	[14,238,239,240]
Community-level tolerance	Combination strategies	AMP + adjuvant or AMP cocktails	AMP-enzyme inhibitor pairs	Lower resistance emergence risk	[8,241,242]
Environmental persistence	Built-in degradability	Triggered lability (salinity-, light-sensitive)	Marine-adapted AMPs	Reduced ecological accumulation	[243,244]

## 7. Strategies to Detect and Disarm the Anti-AMP Resistome

Understanding the breadth and evolution of anti-AMP resistance requires a comprehensive strategy that integrates environmental surveillance, functional screening, molecular profiling, and therapeutic innovation. These efforts must parallel those applied to antibiotic resistance, yet be tailored to the unique properties of AMPs [225,245].

### 7.1. Metagenomic Surveillance of Resistance Determinants

Expanding existing antimicrobial resistance gene (ARG) surveillance frameworks to include AMP-specific resistance genes is essential [246]. Metagenomic profiling of diverse habitats, such as soil, aquatic systems, animal microbiomes, and hospital effluents, can reveal the environmental reservoirs and dissemination potential of genes encoding AMP-inactivating enzymes, efflux transporters, and envelope-modifying proteins [131]. Notably, several of these genes reside on mobile genetic elements, indicating their capacity for horizontal gene transfer into clinically relevant strains. A dedicated anti-AMP resistance gene database, akin to CARD or ResFinder, would enable preclinical assessment of environmental risks for AMP candidates, supporting proactive stewardship and design [247].

### 7.2. Functional Metagenomics to Uncover Novel Resistance Elements

Sequence-based annotation may overlook resistance genes lacking known homology. Functional metagenomic screening circumvents this by cloning environmental DNA into a model host and selecting for clones that survive AMP exposure [247]. This approach has identified previously uncharacterized resistance factors, including hypothetical proteins that confer partial protection against peptides like cecropin and indolicidin. Libraries derived from AMP-rich environments, such as marine sediments or insect guts, are especially valuable [248,249]. The discovery of novel effector genes through this method provides both early warning and mechanistic insight into resistance pathways that may not be evident from genomic data alone [250].

### 7.3. Inducible Transcriptomic and Proteomic Profiling

Many AMP resistance mechanisms are conditionally expressed in response to peptide exposure [251]. Transcriptomic and proteomic analyses of key pathogens under subinhibitory AMP stress can identify inducible operons, regulatory nodes, and metabolic adjustments that comprise the active resistome [252]. This approach has revealed upregulation of stress response genes, membrane remodeling pathways, and even central metabolic regulators not traditionally associated with antimicrobial resistance [253]. Mapping these responses across peptide classes (e.g., α-helical vs. cyclic structures) can distinguish generalist defenses from structure-specific adaptations, guiding both mechanistic studies and rational AMP design [254].

### 7.4. Tracking Co-Selection and Mobile Genetic Elements

Resistance to AMPs often co-occurs with antibiotic and metal resistance on shared mobile elements [255]. Monitoring the genetic linkage between AMP resistance genes and classical ARGs, especially on integrative conjugative elements and multidrug plasmids, provides insight into potential co-selection dynamics. For example, polymyxin resistance genes such as mcr can be co-localized with determinants that modulate susceptibility to HDPs, and selection by one compound class can maintain resistance to others. Wastewater and aquaculture settings, in particular, are hotspots for such co-resistance [256]. Surveillance of these mosaic elements is critical for anticipating the environmental emergence of multidrug-resistant strains that compromise AMP efficacy [257].

### 7.5. Exploiting Resistome Insights for Therapeutic Intervention

Mapping the anti-AMP resistome offers opportunities to develop resistance-disarming strategies. Central regulators such as two-component systems (e.g., PhoPQ, ParRS) or AMP-inducible proteases are potential targets for adjuvant therapies. Small-molecule inhibitors or peptide co-therapies that block these regulators can sensitize bacteria to AMPs [258]. Furthermore, functional screening of resistance proteins may reveal vulnerabilities, such as binding proteins or modifying enzymes, that can be circumvented by structural alterations in AMP design. By integrating resistome knowledge into the early stages of drug development, researchers can preemptively address liabilities and construct AMP candidates with built-in resistance evasion [259].

### 7.6. Toward a Proactive Resistome-Monitoring Framework

**Figure 5:**
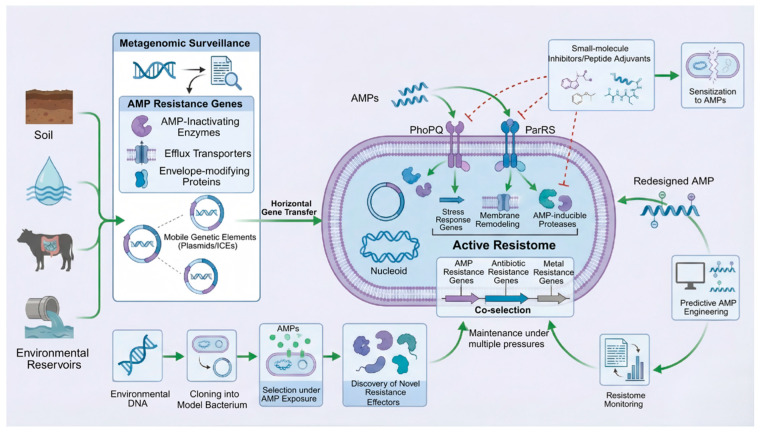
Strategies to detect, monitor, and disarm the anti-AMP resistome. Integrated framework linking resistome surveillance with resistance-aware therapeutic development. Environmental and host-associated reservoirs are profiled to identify genetic and functional determinants of AMP resistance, including envelope remodeling, efflux, and proteolytic inactivation. These insights inform rational AMP design and adjunctive strategies aimed at anticipating, monitoring, and counteracting resistance during therapeutic deployment. Created with BioRender.

Future AMP development requires not only high-throughput screening for activity but also parallel risk assessment of resistance emergence (Figure 5) [260]. This entails systematic environmental surveillance, functional dissection of resistance pathways, and incorporation of resistome awareness into computational modeling and drug screening workflows [261]. Ultimately, the goal is to shift from reactive detection of resistance after clinical failure to predictive, resistance-informed AMP engineering [262].

### 7.7. Experimental and Translational Limitations in Studying AMP Resistance

Despite substantial mechanistic insight into AMP resistance, important experimental limitations must be acknowledged. Much of the current evidence derives from in vitro assays conducted under simplified conditions, including planktonic cultures, defined media, and acute peptide exposure. These systems often fail to capture key features of real-world contexts, such as spatial heterogeneity, host-derived matrices, fluctuating peptide concentrations, polymicrobial interactions, and chronic subinhibitory exposure.

In vitro susceptibility testing may therefore underestimate both the prevalence and durability of AMP tolerance, particularly for mechanisms that depend on biofilm formation, regulatory induction, or stress adaptation. Conversely, laboratory evolution experiments performed under constant and high-peptide pressure may overestimate the likelihood of stable resistance emergence compared with host-associated or environmental settings, where selective forces are intermittent and multifactorial.

Additional biases arise from the widespread use of model organisms and reference strains that may not reflect the genetic and phenotypic diversity of clinical or environmental populations. Opportunistic pathogens encountered in aquaculture, chronic infections, or biofilm-associated niches often experience complex selective landscapes that are poorly approximated by standard laboratory models. Bridging this gap will require experimental frameworks that integrate host-mimetic conditions, multispecies communities, long-term exposure regimes, and environmental parameters.

Recognizing these limitations is essential for translating mechanistic findings into realistic risk assessment and therapeutic design. Without careful consideration of model-dependent biases, the contribution of the anti-AMP resistome to treatment failure, immune evasion, and environmental persistence may be systematically underestimated.

## 8. Clinical and One Health Implications

Within a One Health framework, which integrates human, animal, and environmental health, antimicrobial resistance is understood as an interconnected phenomenon that transcends clinical boundaries. In this context, AMPs and their resistance determinants cannot be considered in isolation, as selective pressures and adaptive traits emerging in environmental and animal-associated microbiomes can directly influence resistance dynamics relevant to human infections. Applying a One Health perspective to AMP resistance underscores how HDPs, therapeutic peptide deployment, and environmental exposure jointly shape the evolution, persistence, and dissemination of anti-AMP resistomes across ecological interfaces.

AMPs are increasingly recognized as promising therapeutic and prophylactic agents [263]. However, the existence of a pervasive anti-AMP resistome challenges their long-term viability. Addressing this issue requires a comprehensive understanding of resistance dynamics across clinical and environmental contexts under a One Health framework [264].

### 8.1. Rapid Emergence of Resistance in Clinical Settings

**Figure 6:**
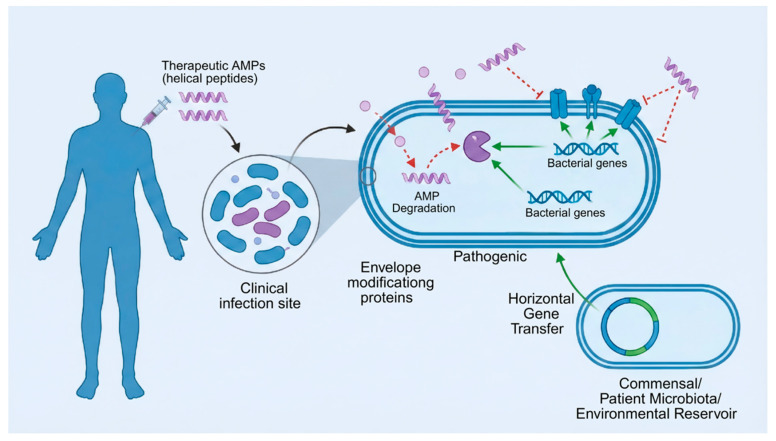
Pre-existing resistomes facilitate rapid AMP resistance emergence. Conceptual illustration of how resistance determinants present in commensal, environmental, or patient-associated microbiota can be mobilized to compromise therapeutic AMP efficacy. Created with BioRender.

AMPs introduced into medical use without prior assessment of environmental or microbiome-based resistance reservoirs risk immediate compromise (Figure 6) [265]. Resistance determinants, such as AMP-degrading enzymes or envelope-modifying genes, may already exist in environmental or commensal bacteria, even before clinical deployment [266]. Analogous to the *mcr-1*–polymyxin paradigm, where agricultural exposure predated clinical resistance outbreaks, AMP failure could arise not from de novo mutation but from horizontal acquisition of pre-adapted mechanisms [267]. Therefore, baseline resistome mapping of patient microbiota, especially in infection-prone populations, is essential prior to AMP approval. Such profiling could inform dosing strategies or contraindications for subpopulations with high innate AMP tolerance [268].

Moreover, Mechanistic insights into the anti-AMP resistome acquire particular significance when considered in clinically relevant contexts. Chronic infections and device-associated biofilms provide archetypal environments in which AMP resistance mechanisms directly compromise therapeutic efficacy. In chronic wound infections and cystic fibrosis airways, persistent biofilm growth, high protease activity, and sustained exposure to HDPs select for bacterial populations with enhanced envelope remodeling, efflux capacity, and peptide-degrading activity, thereby reducing the effectiveness of both endogenous AMPs and peptide-based therapeutics.

Similar challenges arise in medical device-associated infections, including catheters, prosthetic joints, and implanted sensors, where surface-associated biofilms impose diffusion barriers and maintain subinhibitory peptide concentrations. Under these conditions, inducible tolerance mechanisms can be rapidly engaged, while prolonged exposure favors the stabilization of heritable resistance traits. Experimental and preclinical studies have demonstrated that AMP efficacy against biofilm-embedded bacteria is markedly reduced compared with planktonic counterparts, even when minimal inhibitory concentrations appear favorable in standard assays.

Combination therapies offer a promising strategy to counteract these limitations. Several preclinical studies have shown that AMPs can potentiate antibiotic activity by transiently increasing membrane permeability, while antibiotics can suppress bacterial growth states that favor AMP tolerance. However, the success of such combinations critically depends on an informed understanding of the underlying resistome. If AMP-induced envelope remodeling or efflux is already active, synergistic effects may be attenuated or lost. These observations underscore the necessity of integrating resistome-aware design and context-specific testing when advancing AMP-based therapies toward clinical application.

### 8.2. Environmental Stewardship and Agricultural Use

The application of AMPs in agriculture, aquaculture, and animal husbandry presents additional concerns [269]. Use in livestock feed, fish farming, or plant pathogen control may expose environmental microbiota to selective pressure, driving the enrichment and dissemination of resistance genes [270]. These genes, once established in soil or aquatic ecosystems, can transfer to human pathogens via food chains, occupational exposure, or contaminated water [271]. Regulatory oversight must extend beyond efficacy to include environmental risk assessments. AMP deployment should be accompanied by longitudinal monitoring of resistome shifts in adjacent ecosystems, particularly in high-use settings [272].

Marine aquaculture operates within a high-background peptide landscape, since cultured fish and invertebrates continuously secrete HDPs into mucus and rearing water [273]. When peptide-based products are added through feed, immersion, coatings, or localized treatment, they can amplify selection within net-associated biofilms, recirculating biofilters, and receiving sediments, with potential consequences for non-target microbial functions that sustain water quality [274,275]. We therefore recommend that marine AMP deployment be paired with monitoring of biofilm and sediment communities, with explicit tracking of anti-AMP determinants alongside conventional antibiotic resistance markers [273,275].

### 8.3. Integration of Resistome Evaluation into Drug Development

Future AMP pipelines must incorporate standardized resistome risk assessments [276]. These should include in vitro screening against resistance gene-expressing panels, evaluation of cross-resistance to innate immune peptides, and community-level exposure assays to detect emergent tolerance. In silico analyses of metagenomic data can identify whether AMP-targeting genes are frequently co-located with mobile elements or ARGs, suggesting high transferability [277]. When high-risk features are detected, mitigations, such as adjuvant co-administration or indication restriction, should be preemptively planned [278]. Regulatory agencies should treat AMP approval with the same caution and pre-market resistance scrutiny as new antibiotics.

### 8.4. Patient-Specific Microbiome Considerations

Resistance to AMPs may vary across patient populations. Individuals with chronic infections or prior antibiotic exposure often harbor microbiota enriched in resistance elements [279]. For example, cystic fibrosis patients frequently host pathogens with AMP-adaptive traits due to prolonged inflammation. Microbiome-informed precision therapy, similar in principle to genotypic resistance profiling for antibiotics, may facilitate patient stratification and guide AMP selection or dosing strategies [280]. Conversely, indiscriminate AMP use in low-risk individuals, such as nasal decolonization or oral hygiene products, may inadvertently enrich for resistant flora, seeding future transmission or opportunistic infections [281].

### 8.5. Impact on Host Immunity and Immunopathology

**Figure 7:**
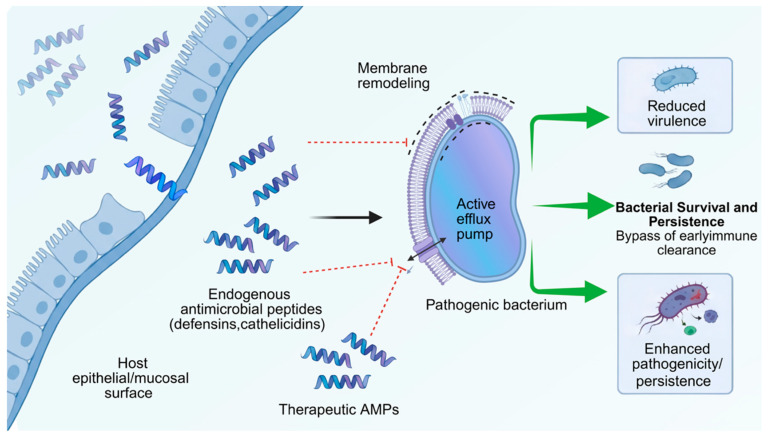
Impact of anti-AMP resistance on host–pathogen interactions. Overview of how bacterial AMP resistance mechanisms influence interactions with host innate immune defenses and infection outcomes. Created with BioRender.

AMP resistance is not confined to therapeutic applications; it may also compromise the effectiveness of endogenous innate immune defenses (Figure 7) [282]. Cationic peptides such as defensins and cathelicidins are critical first-line defenses in mucosal and epithelial surfaces [283]. Resistance mechanisms acquired through environmental AMP exposure may confer cross-protection against these endogenous peptides, allowing pathogens to bypass early immune clearance. Emerging data suggest overlap in resistance pathways between therapeutic AMPs and immune peptides, including membrane remodeling and efflux activation [284]. While certain resistance traits may attenuate bacterial virulence (e.g., LPS loss increasing neutrophil susceptibility), others enhance pathogenicity and persistence. Thus, AMP resistance has direct implications for host–pathogen dynamics and must be considered in immunological risk modeling [285,286,287].

#### Strategic Recommendations

To mitigate these risks, a series of coordinated actions is warranted:

Surveillance: Initiate routine tracking of AMP resistance determinants in clinical, agricultural, and environmental settings, including retrospective screening of archived isolates.

Guideline development: Establish evidence-based frameworks for AMP use in non-clinical settings to avoid unregulated proliferation. Lessons from biocides such as triclosan underscore the risk of unchecked use.

Combination therapy design: Co-formulation of AMPs with antibiotics or resistance inhibitors may offer synergistic effects and reduce resistance emergence. Heterogeneous AMP cocktails targeting distinct pathways could similarly enhance robustness.

One Health coordination: Implement unified regulatory strategies spanning human, animal, and environmental health sectors. New AMP approvals should require environmental impact assessments and stewardship plans.

In conclusion, the promise of AMPs must be balanced against the reality of microbial adaptability. The anti-AMP resistome underscores that resistance is not hypothetical but evolutionarily grounded and ecologically widespread. Integrating resistome awareness into AMP development and policy is not optional—it is imperative. Only by doing so can we preserve the utility of AMPs and uphold the integrity of both therapeutic and innate immune defense systems.

## 9. Outlook

As AMPs transition from experimental tools to real-world applications across medicine, agriculture, and environmental settings, the urgency to anticipate and mitigate resistance cannot be overstated. Future research must prioritize high-resolution mapping of the anti-AMP resistome across ecological compartments, leveraging metagenomics, single-cell transcriptomics, and functional screening to reveal hidden reservoirs and transfer routes. A particular challenge lies in disentangling constitutive versus inducible resistance mechanisms, as many microbial defenses are environmentally modulated and context-specific. Novel frameworks, such as ecological network modeling or systems pharmacology-informed AMP design, may enable prediction of resistance emergence before clinical failure.

Technologically, AMP development must evolve beyond activity-centric metrics. Structure–resistome relationships, collateral immune interactions, and impact on host-associated microbiomes require equal consideration. The integration of artificial intelligence in peptide design offers promise but demands rigorous validation against real-world microbial diversity and biofilm contexts [288]. Regulatory systems should incorporate environmental resistome risk assessments alongside traditional efficacy and safety criteria.

From a One Health perspective, international coordination is needed to establish global AMP stewardship guidelines. This includes tracking AMP usage, resistance gene spread, and ecological consequences in diverse sectors—from aquaculture to hospitals. Only through such cross-disciplinary foresight can AMPs fulfill their therapeutic potential without repeating the failures of antibiotic overuse.

## 10. Conclusions

AMPs represent a powerful yet biologically familiar class of therapeutics. Their ancient evolutionary roots and broad-spectrum activity make them attractive alternatives or complements to traditional antibiotics. However, this very familiarity has also shaped a diverse and entrenched resistome within microbial communities. As this review highlights, AMP resistance is not a future threat—it is a present reality embedded in environmental, clinical, and commensal microbiota.

Recognizing the complexity of anti-AMP resistance is essential for responsible deployment. The resistome spans genetic, physiological, structural, and ecological dimensions, and it cannot be circumvented by design ingenuity alone. Rather, proactive surveillance, contextualized deployment, combination therapies, and environmental safeguards must be integral to AMP development pipelines.

By embedding resistome awareness into every stage of AMP research and application, we can extend the lifespan of these promising molecules. The future of AMPs depends not only on how well they kill, but on how wisely we use them.

## Figures and Tables

**Table 1:** Conceptual framework distinguishing the anti-AMP resistome from related resistance and tolerance paradigms.

Concept	Definition	Genetic Basis	Reversibility	Dominant Selective Pressure	Representative Features
Intrinsic resistance	Constitutive insensitivity inherent to a bacterial species	Encoded in core genome	No	None required	Low membrane permeability, absence of targets
Tolerance	Transient survival without altered susceptibility	None	Yes	Acute stress	Growth arrest, dormancy
Adaptive resistance	Inducible, reversible phenotypes triggered by environmental cues	Regulatory	Yes	Repeated or sublethal stress	Two-component systems, envelope stress responses
Antibiotic resistome	Genetic repertoire conferring resistance to small-molecule antibiotics	Often heritable	Variable	Antibiotics	Enzymatic degradation, target modification
Anti-AMP resistome	Integrated network of genetic, regulatory, and physiological mechanisms enabling survival under AMP pressure	Genetic, regulatory, and physiological	Variable	HDPs and AMP exposure	Surface charge modulation, membrane remodeling, proteolysis, efflux, biofilm buffering

## Data Availability

All data reported in this paper will be shared by the lead contact [Pengfei Cui, e-mail: cuipengfei@ouc.edu.cn] upon reasonable requests. Any additional information required to reanalyze the data reported in this article is available from the lead contact upon request.
